# Copper ions inhibit *Streptococcus mutans–Veillonella parvula* dual biofilm by activating *Streptococcus mutans* reactive nitrogen species

**DOI:** 10.1186/s12903-023-02738-0

**Published:** 2023-01-28

**Authors:** Zhang Yun, Liu Xianghong, Gao Qianhua, Du Qin

**Affiliations:** 1 State Key Laboratory of Oral Diseases and National Clinical Research Center for Oral Diseases, West China School of Stomatology, Sichuan University, Chengdu, 610041 Sichuan China; 2 Department of Pediatric Dentistry, West China Hospital of Stomatology, Sichuan University, Chengdu, 610041 Sichuan China; 3 Department of Stomatology, Sichuan Provincial People’s Hospital, University of Electronic Science and Technology of China, Chengdu, 610072 China

**Keywords:** Copper ion, *Streptococcus mutans*, *Veillonella parvula*, Biofilm, *copYAZ*

## Abstract

**Background:**

To investigate the inhibition mechanism of copper ions on *Streptococcus mutans–Veillonella parvula* dual biofilm.

**Methods:**

*S. mutans–V. parvula* dual biofilm was constructed and copper ions were added at different concentrations. After the biofilm was collected, RNA-seq and qRT-PCR were then performed to get gene information.

**Results:**

The coculture of *S. mutans* and *V. parvula* formed a significantly better dual biofilm of larger biomass than *S. mutans* mono biofilm. And copper ions showed a more significant inhibitory effect on *S. mutans–V. parvula* dual biofilm than on *S. mutans* mono biofilm when copper ions concentration reached 100 µM, and copper ions showed a decreased inhibitory effect on *S. gordonii–V. parvula* dual biofilm and *S. sanguis–V.parvula* dual biofilm than on the two mono biofilms as the concentration of copper ions increased. And common trace elements such as iron, magnesium, and zinc showed no inhibitory effect difference on *S. mutans–V. parvula* dual biofilm. The RNA-seq results showed a significant difference in the expression of a new ABC transporter *SMU_651c*, *SMU_652c*, *SMU_653c*, and *S. mutans* copper chaperone *copYAZ*. *SMU_651c*, *SMU_652c*, and *SMU_653c* were predicted to function as nitrite/nitrate transporter-related proteins, which suggested the specific inhibition of copper ions on *S. mutans–V. parvula* dual biofilm may be caused by the activation of *S. mutans* reactive nitrogen species.

**Conclusions:**

*Streptococcus mutans* and *Veillonella parvula* are symbiotic, forming a dual biofilm of larger biomass to better resist the external antibacterial substances, which may increase the virulence of *S. mutans*. While common trace elements such as iron, magnesium, and zinc showed no specific inhibitory effect on *S. mutans–V. parvula* dual biofilm, copper ion had a unique inhibitory effect on *S. mutans*–*V. parvula* dual biofilm which may be caused by activating *S. mutans* RNS when copper ions concentration reached 250 µM.

**Supplementary Information:**

The online version contains supplementary material available at 10.1186/s12903-023-02738-0.

## Background

Dental caries is one of the most common oral diseases endangering human health. Its occurrence and development are closely related to dental biofilm [1]. *Streptococcus mutans (S. mutans)* is a major causative bacterium of dental caries. Crucial to *S. mutans’* cariogenicity is the ability to attach to the tooth surface and interact with other bacteria to form a tenacious, well-structured biofilm [2, 3]. *Veillonela parvula (V. parvula*) is a gram-negative oral commensal [4, 5], which has been reported to help *S. mutans* to form a thicker biofilm and to have better protection against external bactericidal substances [6–8].

Copper is indispensable for maintaining the normal physiological functions of the human body. Its physiological concentration in saliva ranges from 0.2 to 7.05 mg L^−1^ and high concentrations of copper ions can be used as bacteriostats [9]. Werner’s research had shown that copper ions could significantly inhibit the growth of *S. mutans* [10]. Furthermore, the abilities of *S. mutans* to form biofilms and develop genetic competence were impaired under copper stress [11]. It has also been reported that a higher concentration of copper ions in the oral cavity may reduce the incidence of dental caries [5]. Considering the antibacterial properties of copper ions, it has been used by scientists in various oral materials to prevent the occurrence and development of oral diseases such as caries and periodontal diseases [12, 13]. But the antibacterial mechanism is not fully understood.

This study aimed to investigate the inhibitory effect of copper ions on the *Streptococcus mutans* mono biofilm and *S. mutans*–*V. parvula* dual biofilm, compare the inhibitory ability difference of copper ions on these two kinds of biofilms, and explore the mechanism of the inhibitory effects of copper ions on the *S. mutans*–*V. parvula* dual biofilm.

In this study, copper ions were found to have a better inhibitory effect on *S. mutans–V. parvula* dual biofilm than on *S. mutans mono biofilm*. The result of RNA-seq showed that copper ions may exhibit bacteria inhibitory effects by activating *S. mutans* reactive nitrogen species (RNS). But the mechanism still needs further clarification.

## Material and methods

### Bacterial strains and growth conditions

*S. mutans* strain UA159, *S. gordonii* strain DL 1, *S. sanguis* strain SK 36, *and V. parvula* PK1910 were used in this study. Biofilms were formed using a method similar to that of S.S. Garcia et al. [9]. In brief, *S. mutans/S. gordonii/S. sanguis* cultured overnight was diluted into fresh THB and grown to the exponential phase. Cultures were diluted at a proper ratio of 1:100 into 10 ml of pre-warmed THB containing 1% sucrose as the carbohydrate source to form the *S. mutans/S. gordonii/ S. sanguis* mono biofilm [14]. *V. parvula* cultured overnight was diluted into fresh THL (THB + 0.1% Sodium Lactate) and grown to the exponential phase. Cultures were diluted at a proper ratio of 1:20 for *V. parvula* and 1:100 for *S. mutans/S. gordonii/S. sanguis* into 10 ml prewarmed THL + 1% sucrose to form the *S. mutans/S. gordonii/S. sanguis–V. parvula* dual biofilm. Biofilms were grown statically for 18 h in 96-well polystyrene plates with three repeats.

### Different concentrations of metal ions

Metal ions at different concentrations were added to the *S. mutans–V. parvula* dual biofilm. Iron (FeSO_4_) and magnesium (MgCl_2_, MgSO_4_) were added to the culture at concentrations of 100 µM, 500 µM, 1000 µM, and 2000 µM respectively. For zinc ions (ZnSO_4_), concentrations of 10 µM, 50 µM, 100 µM, 250 µM, 500 µM, 750 µM, 1000 µM, 2000 µM and 3000 µM were respectively added. As for copper ions (CuSO_4_), concentrations of 10 µM, 50 µM, 100 µM, 250 µM, 500 µM, 750 µM, and 1000 µM were respectively added.

### Biomass measurement of biofilms

To measure biomass, loosely adhered bacteria cells were gently washed off and the biofilms were stained with 0.1% crystal violet for 15 min and solubilized in 30% acetic acid. Biomass was quantified using OD562.

### Transcriptome analysis by RNA-seq

Mono- and dual-species cultures were prepared similarly to those before. Biofilms were grown statically for 18 h in 6-well polystyrene plates overnight, and loosely adhered bacteria cells were gently washed off and the biofilms were scratched and harvested by centrifugation and frozen at − 80 °C until use. RNA was isolated by TRIzol® Reagent, and rRNA was removed. RNA-seq was performed by Illumina Hiseq4000.

### QRT-PCR

To validate the RNA-Seq data, quantitative real-time PCR (qRT-PCR) was used to measure the changes in the expression of selected mRNA. The first cDNA was synthesized from 1 μg of purified RNA by the Bio-Rad iScript cDNA synthesis kit (Bio-Rad Laboratories, Inc., Hercules, CA, United States). And quantitative amplification condition was made with Bio-Rad iTaq Universal SYBR Green Supermix and Bio-Rad CFX96 system (Bio-Rad Laboratories, Inc.). To determine the relative amount of cDNA molecules, standard curves were used for each primer (Additional file 1: Table S1). Meanwhile, relative expression was calculated by normalizing the validated reference gene gyrA transcripts [15, 16]. The publication of qRT-PCR experiments (MIQE) guidelines was followed to control the quality of the data as well as analyze the information [17].

Three separated approaches were used to calculate fold changes and significant differences in gene expression between growth conditions: DEseq, edgeR, and limma [18–20], as implemented in the R/Bioconductor package metaseqR [21]. We assigned genes GO terms using Blast2GO v.2.5.0 [22]. And Fisher’s exact tests were used to assess the relative enrichment of GO terms [23]. The test was performed using the Gossip statistical package. According to previous research, the false discovery rate (FDR) of 0.05 was used to correct for multiple hypothesis testing [24].

A KEGG pathway impact analysis was performed by the software package Pathway-Express as implemented in the R/Bioconductor package ROntoTools [25]. Also, the FDR procedure of 0.05 was used to correct for multiple hypothesis testing [24].

### Statistics

The biomass difference between co-cultured and mono-cultured biofilms without adding copper ions was analyzed through two independent samples t-test and elsewhere t-test. The ratio of “OD562 value without copper ions’ addition minus OD562 value with copper ions’ addition at different concentrations” to “OD562 value without copper ions’ addition” was taken as the inhibition rate of biofilms to represent the inhibitory effect on biofilms. The difference in the inhibition rate of biofilms of copper ions at different concentrations between co-cultured and mono-cultured biofilms was analyzed through two independent samples t-test. The difference in gene expression before and after adding copper ions was analyzed through paired sample t-test. A *P* value < 0.05 was considered significant.

## Results

### Copper ions had a unique inhibitory effect on *S. mutans*–*V. parvula* dual biofilm

**Fig. 1:**
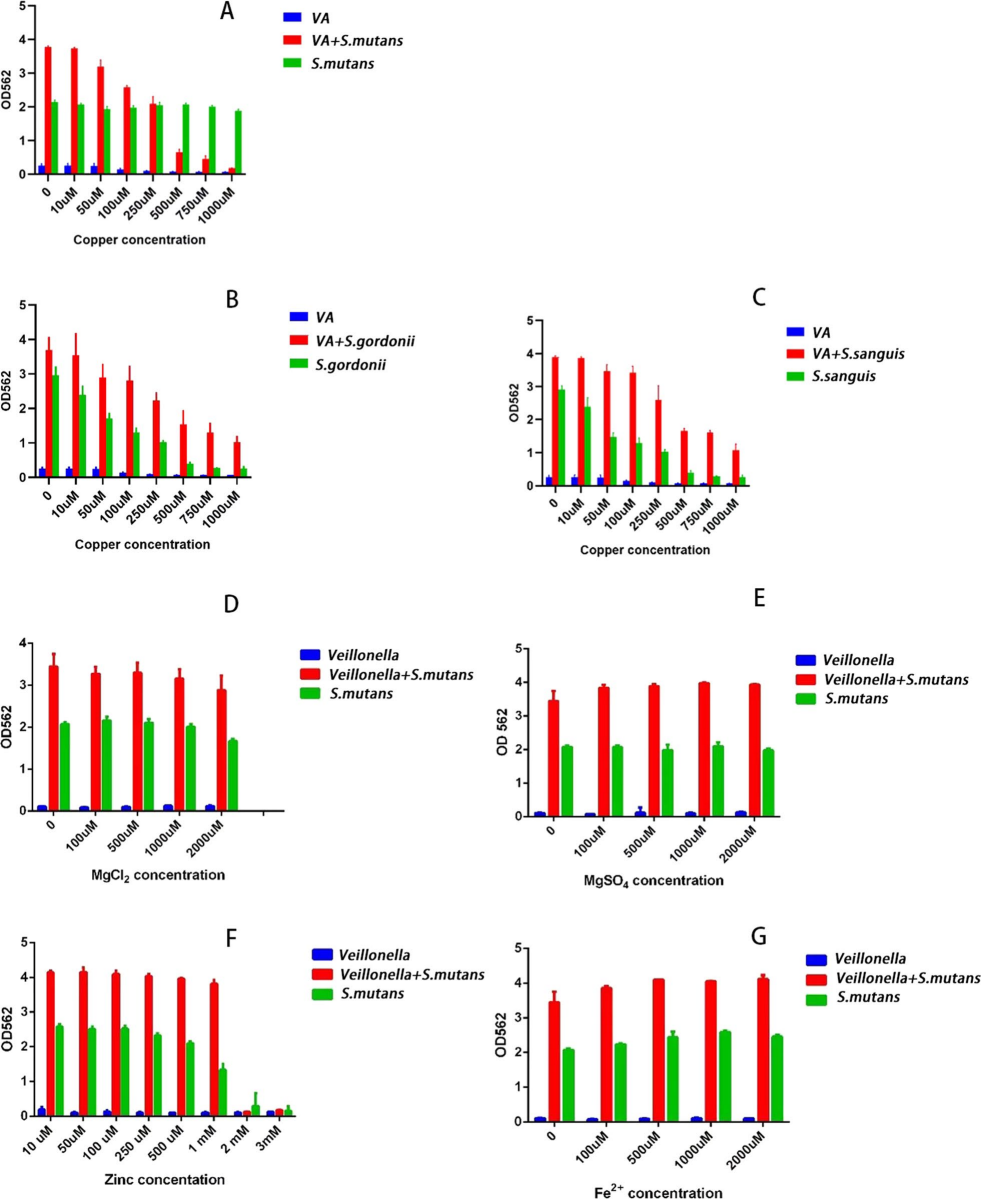
Inhibitory effect of copper ions, magnesium ions, zinc ions, and ferrous ions on biofilm. **A** Copper ions had a unique inhibition on *S. mutans–V. parvula* dual biofilm (*P* < 0.05). **B**, **C** When no copper ions were added, there was a statistically larger biomass of *S. sanguis–V. parvula* dual biofilm than *S. sanguis* mono biofilm (*P* < 0.05), while the biomass of *S. gordonii–V. parvula* dual biofilm and *S. gordonii* mono biofilm had no statistical differences (*P* > 0.05). And copper ions showed a decreased inhibitory effect on these two dual biofilms than on the two mono biofilms as the concentration of copper ions increased (*P* < 0.05). **D**–**G** Magnesium ions, zinc ions, and ferrous ions were added, and these trace elements showed no specific inhibitory effect on *S. mutans–V. parvula* dual biofilm (*P* > 0.05)

Co-culture of *S. mutans* and *V. parvula* formed a significantly better biofilm than *S. mutans* mono biofilm when no copper ions were added (*P* < 0.05). And at the copper ions concentrations of 250 µM or below, the growth of *S. mutans–V. parvula* dual biofilm was significantly better than the *S. mutans* mono biofilm for the biofilm biomass was statistically greater than that of *S. mutans* mono biofilm (*P* < 0.05)*.* While the inhibitory effect of copper ions on the dual biofilm was significantly better than that on the *S. mutans* mono biofilm when the concentration of copper ions reached 100 µM (*P* < 0.05) (Fig. 1A).

### Copper ions’ inhibition effect on *S. gordonii*–*V. parvula* dual biofilm and *S. sanguis*–*V. parvula* dual biofilm

When no copper ions were added, there was a statistically larger biomass of *S. sanguis-V. parvula* dual biofilm than *S. sanguis* mono biofilm (*P* < 0.05), while the biomass of *S. gordonii-V. parvula* dual biofilm and *S. gordonii* mono biofilm had no statistical differences (*P* > 0.05). And copper ions showed decreased inhibitory effect on these two dual biofilms than on the two mono biofilms as the concentration of copper ions increased (*P* < 0.05) (Fig. 1B, C).

### The inhibitory effect of common trace elements on *S. mutans*–*V. parvula* dual biofilm

Common trace elements such as iron, magnesium, and zinc were added, and these trace elements showed no specific inhibitory effect on *S. mutans–V. parvula* dual biofilm (*P* > 0.05) (Fig. 1D–G).

### RNA-seq

A total of 29.6, 38.7, 33.3 and 26.2 million reads were obtained from the *S. mutans* mono biofilm, *S. mutans–V. parvula* dual biofilm, *S. mutans–V. parvula* dual biofilm under 250 μM copper ions, *S. mutans–V. parvula* dual biofilm under 500 μM copper ions using sequencing respectively. RNA-seq results of the *S. mutans–V. parvula* dual biofilm revealed 179 genes up-regulated compared to *S. mutans* mono biofilm, involving multi biological processes, such as the biological process, cellular components, and molecular functions (Additional file 1: Table S2).

RNA-seq results of the *S. mutans–V. parvula* dual biofilm with 250 μM copper ions revealed that 723 genes changed compared to which without copper ions’ addition, involving multi biological processes, such as the biological process, cellular components, and molecular functions (Additional file 1: Table S3).

RNA-seq results of the *S. mutans–V. parvula* dual biofilm with 500 μM copper ions revealed that 34 genes changed compared to which with 250 μM copper ions, involving multi biological processes, such as the biological process, cellular components, and molecular functions (Additional file 1: Table S4).

### Differences in gene expression before and after adding copper ions

Copper transport system *copY*, *copA*, *copZ*, and nitrate/nitrite metabolic pathway: *SMU_651c*, *SMU_652c*, *SMU_653c*.

*CopY, copA, copZ and SMU_651c, SMU_652c, SMU_653c* showed no significant difference between *S. mutans* mono biofilm group and *S. mutans–V. parvula* dual biofilm group when no copper ions were added (*P* > 0.05) (Table 1).

**Table 1:** The gene expression difference between *S. mutans* mono biofilm and *S. mutans–V. parvula* dual biofilm before copper ions’ addition

Seq_id	sm_count	sm_vp_count	sm_fpkm	sm_vp_fpkm	log2FC (sm_vp/sm)	*P* value	FDR	Significant	Regulate
*SMU_651c*	674.443	842.485	74.9465	93.1269	0.31	9.49E−01	9.98E−01	NS	Up
*SMU_652c*	347.107	311.042	58.6953	52.1764	− 0.17	5.14E−01	8.37E−01	NS	Down
*SMU_653c*	360.289	228.988	42.2153	26.6818	− 0.66	1.68E−01	5.20E−01	NS	Down
*CopZ*	547.024	675.515	4736.76	4960.58	0.07	9.68E−01	9.98E−01	NS	Up
*CopA*	81,090.2	71,135.1	3641.4	3187.54	− 0.19	4.77E−01	8.19E−01	NS	Down
*CopY*	8326.18	10,783.4	3291.1	4183.63	0.35	8.83E−01	9.90E−01	NS	Up

NS *P* > 0.05

*CopA, copZ, SMU_651c, SMU_652c, and SMU_653c* were significantly up-regulated in the *S. mutans–V. parvula* dual biofilm group with 250 µM copper ions (while *copY* was up-regulated, although with no significant difference) (*P* < 0.05) (Table 2). Consistent with the previous result, the dual biofilm was significantly inhibited when copper ions’ concentration reached 250 µM.

**Table 2:** The gene expression difference of *S. mutans–V. parvula* dual biofilm before and after adding 250 µM copper ions

seq_id	sm_vp_count	sm_vp250_count	sm_vp_fpkm	sm_vp250_fpkm	log2FC (sm_vp200/sm_vp)	*P* value	FDR	Significant	Regulate
*SMU_651c*	842.485	20,727	93.1269	2287.36	4.62	2.04E−09	7.61E−08	*	Up
*SMU_652c*	311.042	14,031.6	52.1764	2347.88	5.49	7.53E−12	8.79E−10	*	Up
*SMU_653c*	228.988	13,989.2	26.6818	1627.2	5.93	4.25E−13	8.82E−11	*	Up
*CopZ*	675.515	2415.5	4960.58	17,156.7	1.79	9.56E−03	2.80E−02	*	Up
*CopA*	71,135.1	288,381	3187.54	12,913.6	2.02	4.26E−03	1.48E−02	*	Up
*CopY*	10,783.4	25,701.5	4183.63	9914.07	1.24	7.83E−02	1.51E−01	NS	Up

NS P > 0.05; *P < 0.05

*After adding 500 µM of copper ions, copY, copA, and copZ* showed no significant difference (*P* > 0.05), while *SMU_651c*, *SMU_652c*, *SMU_653c* showed a significant decrease (*P* < 0.05), which may be caused by massive cell death (Table 3).

**Table 3:** The gene expression difference between *S. mutans–V. parvula* dual biofilm under the copper ions’ concentration of 250 µM and 500 µM

seq_id	sm_vp250_count	sm_vp500_count	sm_vp250_fpkm	sm_vp500_fpkm	log2FC (sm_vp500/sm_vp200)	*P* value	FDR	Significant	Regulate
*SMU_651c*	20,727	3937.95	2287.36	434.257	− 2.4	5.58E−04	3.47E−02	*	Down
*SMU_652c*	14,031.6	2164.41	2347.88	361.763	− 2.7	1.21E−04	9.00E−03	*	Down
*SMU_653c*	13,989.2	2660.31	1627.2	309.201	− 2.4	5.63E−04	3.47E−02	*	Down
*CopZ*	2415.5	1508.67	17,156.7	10,408.6	− 0.72	3.21E−01	1.00E+00	NS	Down
*CopA*	288,381	237,674	12,913.6	10,639.8	− 0.28	6.98E−01	1.00E+00	NS	Down
*CopY*	25,701.5	12,145.2	9914.07	4672.76	− 1.09	1.10E−01	8.96E−01	NS	Down

NS *P* > 0.05; *P < 0.05

### QRT-PCR

**Fig. 2:**
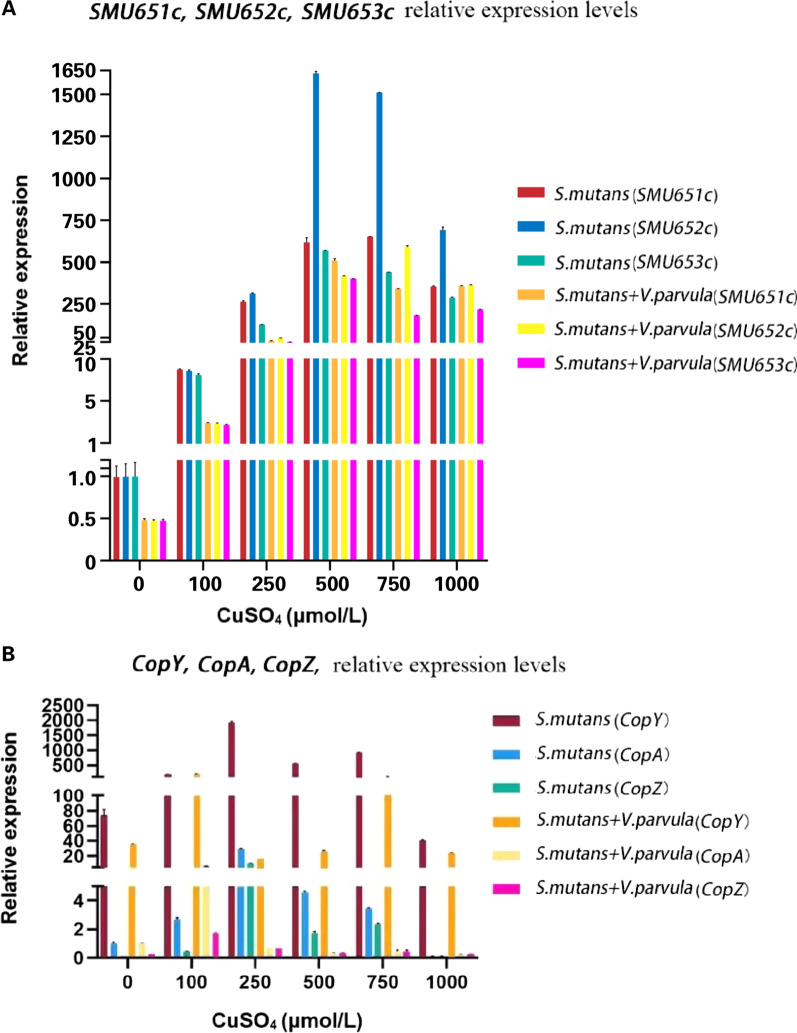
QRT-PCR results. **A** *SMU_651c*, *SMU_652c*, and *SMU_653c* were significantly up-regulated after adding copper ions (*P* < 0.05). **B** *CopA* was up-regulated after adding copper ions in both *S. mutans* mono biofilm and *S. mutans–V. parvula* dual biofilm (*P* < 0.05). *CopY* and *copZ* were up-regulated in the presence of 250 µM copper ions in *S. mutans* mono biofilm (*P* < 0.05)

QRT-PCR results showed that *SMU_651c*, *SMU_652c*, and *SMU_653c* were significantly up-regulated after adding copper ions (*P* < 0.05, Fig. 2A). *CopA* were up-regulated after adding copper ions in both *S. mutans* mono biofilm and *S. mutans–V. parvula* dual biofilm (*P* < 0.05). *CopY* and *copZ* were up-regulated in the presence of 250 µM copper ions in *S. mutans* mono biofilm (*P* < 0.05, Fig. 2B).

## Discussion

The mitis and sanguinis groups, including *S. oralis*, *S. mitis*, *S. gordonii*, and *S. sanguinis*, are the primary colonizers of the tooth surface and are commonly considered as commensals [26]. In particular, communities collected from dentin carious lesions contained notorious acidogenic and aciduric species, including *S. mutans*, *Scardovia wiggsiae*, *Parascardovia denticolens*, and *Lactobacillus salivarius*. In contrast, *S. sanguinis*, *Neisseria species*, and *Leptotrichia species* were found associated with samples collected from healthy sites [27]. In our study, we found that in mono biofilm, copper ions had an inhibitory effect on *S. sanguis* and *S. gordonii* while no significant inhibitory effect on *S.mutans*. Considering the antagonistic effects between these two bacteria and *S. mutans*, it seemed that copper ions could help *S. mutans* compete with the others. But in the complex oral biofilm, the final results require further investigation. We then investigate the copper ions’ effect on dual-biofilm of S. mutans and *V. parvula.*

A lot of studies have shown that *S. mutans* and *V. parvula* are symbiotic. Mashima et al. found that the *S. mutans* and *V. parvula* could form a better biofilm compared to the mono biofilm, and the survival rates of *S. mutans* and *V. parvula* were both increased in the dual biofilm [28]. A study by Kara [29] showed that dual-species biofilms of *S. mutans* and *V. parvula* were less susceptible to antimicrobials, such as chlorhexidine, hydrogen peroxide, erythromycin, and zinc chloride than single-species biofilms of the same microorganisms. Qi’s study showed that *V. parvula* could help *S. mutans* to outcompete *S. gordonii* by improving *S. mutans* carbohydrate utilization and H_2_O_2_ resistance [30]. This study also showed that *S. mutans* and *V. parvula* could symbiosis to form a better biofilm of larger biomass, consistent with previous studies. RNA-seq results showed that in the dual biofilm, 179 gene expressions up-regulated involving in the biological process, cellular components, and molecular functions, suggesting that these processes may be related to thickening dual-biofilm to help improve *S. mutans*’ ability of carbohydrate utilization and H_2_O_2_ resistance. But further studies are still needed to explain the mechanisms.

In this study, copper ions were found to show better inhibitive effect on *S. mutans–V. parvula* dual biofilm than on *S. mutans* mono biofilm. No similar inhibition was found in the other dual biofilm groups such as *S. gordonii-V. parvula* and *S. sanguis-V. parvula* dual biofilms. Other common metal ions such as Fe^2+^, Mg^2+^, and Zn^2+^ were also tested and no such inhibition effect was found, which meant copper ions’ inhibition on *S. mutans–V. parvula* dual biofilm was unique. The RNA-seq results showed that the *S. mutans* copper-binding chaperone *copYAZ* expression increased with the increase of copper ions’ concentration, and at the same time a group of genes that were predicted to express a group of membrane transporters-ABC transporters (ATP-binding cassette transporters) *SMU_651c*, *SMU_652c* and *SMU_653* were also significantly increased, which was confirmed by qRT-PCR. The function of *SMU_651c*, *SMU_652c*, and *SMU_653c* were predicted to be NO/nitrate /nitrite metabolism. Therefore, we speculated that the inhibitory effect of copper ions on *S. mutans–V. parvula* may be related to NO/nitrate/nitrite metabolism.

ABC transporters are a group of transporters that widely exist in bacteria, archaea, and eukaryotes, and their function involved the transmembrane transport of ions, carbohydrates, lipids, and proteins. The newly discovered ABC transporters *SMU_651c*, *SMU_652c*, and *SMU_653c* found in this sequencing were predicted to function as nitrite/nitrate transporter-related proteins (Table 4).

**Table 4:** Function of ABC transporter *SMU_651c*, *SMU_652c*, *SMU_653c*

	Gene ID	Function
*SMU_651c*	1028079	ABC transporter substrate-binding protein
*SMU_652c*	1029588	Nitrate ABC transporter, ATP-binding protein
*SMU_653c*	1028071	Nitrate transport protein, ABC transporter permease

The following ways implicated in the interaction of copper ions and S. mutans have been described in the literature:

1. Copper ions activate *S. mutans* ROS, causing oxidative stress and leading to cell death [11].
2. Copper ions inhibit the expression of biofilm-forming related gene, *GTF* genes (*gtfB*, *gtfC*, *gtfD*) and *GBP* genes (*gbpB*, *gbpC*) of *S. mutans* [11].
3. Copper ions irreversibly suppress the activity of *S. mutans* F-ATPase, affecting the glycolysis ability of bacteria in an acidic environment, resulting in cell death [31].

Since copper ions showed a specific inhibition on *S. mutans–V. parvula* dual biofilm, there might be some other mechanisms for this, combing with the RNA-seq data, we believed that excessive copper ions may activate reactive nitrogen species (RNS), leading to intracellular nitrate/nitrite metabolic disorders.

Since copper ions’ activation to RNS in *S. mutans* has not been reported, we reviewed the literature to confirm the reasonableness of the speculations.

### Excessive copper ions activate RNS in eukaryotes to bring cell damage

It has been widely studied that excessive copper ions release a series of radical ions by redox reaction, and activate inducible nitric oxide synthase (iNOS) to release a large amount of NO, leading to nitrative stress in cells and causing cell death [32]. For example, Cuzzocrea found excessive iNOS expression in a large number of tissues including arteries, liver, and lungs of mice when injected excessive amount of copper ions, which led to excessive nitrotyrosine appearing in tissues, and activated nitrification stress and mediated cell damage [33]. Reddy et al. treated astrocytes with copper ions and found that copper ions mediated intracellular RNS and ROS, resulting in cell damage [34].

### The literature on the activation of RNS by copper ions in prokaryotes was rare

Karreraetal’s study suggested that copper ions exerted the effects as an antibacterial agent in the innate immune system via interaction with reactive nitrogen species [35]. The presence of copper ions in the bacterial cytoplasm could potentiate nitrative stress by causing the uncontrolled release of NO from S-nitroso thiols, which may become toxic to the bacterial cells in the presence or absence of additional host-derived NO.

If it is true that copper ions specifically inhibit *S. mutans–V. parvula* dual biofilm by activating RNS, then in *S. mutans–V. parvula* co-culture, there should be more NO than in *S. mutans* monoculture, which is possible.

*V. parvula* may transfer nitrate into nitrite (reaction (1)), the existence of *S. mutans* furtherly reduces the pH value of the dual biofilm, leading to reaction (2) to produce nitrite, the nitrite forms NO through reaction (3) and (4):

1 $${\text{NO}}^{{{3} - }} + {\text{2H}}^{ + } + {\text{2e}} \to {\text{NO}}^{{{2} - }} + {\text{H}}_{{2}} {\text{O}}$$

2 $${\text{NO}}^{{{2} - }} + {\text{H}}^{ + } \leftrightharpoons {\text{HNO}}_{{2}} \left( {{\text{pKa}}\;{3}.{2}} \right)$$

3 $${\text{2HNO}}_{{2}} \leftrightharpoons {\text{H}}_{{2}} {\text{O}} + {\text{N}}_{{2}} {\text{O}}_{{3}}$$

4 $${\text{N}}_{{2}} {\text{O}}_{{3}} \leftrightharpoons {\text{NO}} + {\text{NO}}_{{2}} .$$

Based on the above reactions, we speculated that the co-culture of *V. parvula* and *S. mutans* may generate exogenous NO, along with endogenous NO activated by copper ions to cause specific killing of dual biofilm cells. The *S. mutans* cultured alone were unable to begin reaction (1) due to the lack of *V. parvula*, so the inhibition of copper ions on *S. mutans–V. parvula* dual biofilm was better than on *S. mutans* mono biofilm.

Due to the positive inhibitory effect of copper ions on *S. mutans–V. parvula* dual biofilm, copper ions could be considered more in the development of oral antimicrobial agents and could be added to multi kinds of dental materials for caries prevention, treatment, and daily oral hygiene maintenance.

## Conclusions

In this study, we found that:

1. *S. mutans* and *V. parvula* were symbiotic, and they could form a better dual biofilm of larger biomass than *S. mutans* mono biofilm. RNA-seq results suggested multi gene expression increases involving biological processes, cellular components, and molecular functions.
2. Copper ions had a unique and better inhibitory effect on *S. mutans–V. parvula* dual biofilm than on *S. mutans* mono biofilm when copper ions concentration reached 100 µM, and copper ions showed decreased inhibitory effect on *S. gordonii-V. parvula* dual biofilm and *S. sanguis-V.parvula* dual biofilm than on the two mono biofilms as the concentration of copper ions increased. And common trace elements such as iron, magnesium, and zinc showed no specific inhibitory effect on *S. mutans–V. parvula* dual biofilm. The RNA-seq results showed that the expression of *S. mutans* copper transporter *copYAZ* genes significantly increased by adding copper ions at the concentration of 250 µM or higher in *S. mutans–V. parvula* dual biofilm and a newly discovered nitrate/nitrite related ABC transporter (*SMU_651c*, *SMU_652c*, *SMU_653c*) expression showed a significant upregulation. Copper ions may inhibit *S. mutans–V. parvula* dual biofilm by activating *S. mutans* RNS.

Further investigation into the antibacterial mechanism of copper ions on *S. mutans–V. parvula* dual biofilm still needs to be carried out and the result may lead to the identification of novel virulence-regulatory pathways.

## Supplementary Information

**Additional file 1. Table S1.** Quantitative reverse transcription polymerase chain reaction primers. **Table S2.** The gene expression difference of *S. mutans* and *S. mutans*–*V. parvula* dual biofilm. **Table S3.** The gene expression difference of *S. mutans*–*V. parvula* dual biofilm with and without 250 μM copper ions. **Table S4.** The gene expression difference of *S. mutans*–*V. parvula* dual biofilm with 250 μM copper ions and 500 μM copper ions.

## Data Availability

The datasets generated and/or analyzed during the current study are submitted to GEO DataSets repository, https://www.ncbi.nlm.nih.gov/geo/query/acc.cgi?acc=GSE222218.

## Abbreviations

*S.** mutans*: *Streptococcus mutans*
*V.** parvula*: *Veillonela parvula*
RNS: Reactive nitrogen species
qRT-PCR: Quantitative real-time PCR
