# Supplementary material for: Copper ions inhibit Streptococcus mutans–Veillonella parvula dual biofilm by activating Streptococcus mutans reactive nitrogen species
Source: BMC Oral Health. 2023 Jan 28;23:48. doi: 10.1186/s12903-023-02738-0 (PMC9883903; doi:10.1186/s12903-023-02738-0)
Supplement: Supplementary file 1 — Additional file 1. Table S1. Quantitative reverse transcription polymerase chain reaction primers. Table S2. The gene expression difference of S. mutans and S. mutans–V. parvula dual biofilm. Table S3. The gene expression difference of S. mutans–V. parvula dual biofilm with and without 250 μM copper ions. Table S4. The gene expression difference of S. mutans–V. parvula dual biofilm with 250 μM copper ions and 500 μM copper ions. [file 12903_2023_2738_MOESM1_ESM.docx]

**Supplemental materials**

| Gene name | Primer sequence（5'-3') |
| --- | --- |
| Sm16srDNA-F | AGCGTTGTCCGGATTTATTG |
| Sm16srDNA-R | CTACGCATTTCACCGCTACA |
| SMU_651c-F | AACCTGCCCTAACTGATCGC |
| SMU_651c-R | AGTCTGCTGCCGAGCTAAAA |
| SMU_652c-F | AGCTGGTCTTGGTGTCATGG |
| SMU_652c-R | AGAAGCAAGATGCAGGCTGA |
| SMU_653c-F | ACCGTATTTAACGCCCAGCA |
| SMU_653c-R | TTGCTCATGTTGCCCTTCCA |

*Supplemental Table1*. Quantitative reverse transcription polymerase chain reaction primers

|  | GO term | No. of up gene  (*S.mutans-V.parvula* dual biofilm  VS  *S.mutans* mono biofilm) | No. of up gene  (*S.mutans-V.parvula* dual biofilm  VS  *S.mutans mono* biofilm) |
| --- | --- | --- | --- |
| Biological process | biological regulation | 6 | 0 |
|  | cellular component organization or biogenesis | 8 | 0 |
|  | cellular process | 31 | 0 |
|  | establishment of localization | 4 | 0 |
|  | localization | 4 | 0 |
|  | metabolic process | 31 | 0 |
|  | regulation of biological process | 6 | 0 |
|  | response to stimulus | 3 | 0 |
|  | signaling | 1 | 0 |
| Cellular component | cell | 14 | 0 |
|  | cell part | 14 | 0 |
|  | extracellular region | 1 | 0 |
|  | macromolecular complex | 8 | 0 |
|  | membrane | 5 | 0 |
|  | membrane-enclosed lumen | 1 | 0 |
|  | membrane part | 4 | 0 |
|  | organelle | 8 | 0 |
|  | organelle part | 3 | 0 |
| Molecular function | binding | 23 | 0 |
|  | catalytic activity | 24 | 0 |
|  | molecular transducer activity | 1 | 0 |
|  | nucleic acid binding transcription factor activity | 1 | 0 |
|  | structural molecule activity | 5 | 0 |

*Supplemental Table2*. The gene expression difference of *S. mutans* and *S. mutans-V. parvula* dual biofilm

|  | GO term | No. of up gene  (*sm-vp 250 vs sm-vp*) | No. of down gene  (*sm-vp 250 vs sm-vp*) |
| --- | --- | --- | --- |
| Biological process | biological regulation | 14 | 4 |
|  | cellular component organization or biogenesis | 6 | 12 |
|  | cellular process | 41 | 50 |
|  | establishment of localization | 4 | 14 |
|  | localization | 4 | 14 |
|  | metabolic process | 50 | 55 |
|  | multi-organism process | 1 | 2 |
|  | regulation of biological process | 14 | 4 |
|  | response to stimulus | 3 | 5 |
|  | single-organism process | 28 | 40 |
|  | immune system process | 1 | 0 |
|  | signaling | 2 | 0 |
| Cellular component | cell | 23 | 32 |
|  | cell part | 23 | 32 |
|  | macromolecular complex | 7 | 17 |
|  | membrane | 4 | 17 |
|  | membrane-enclosed lumen | 1 | 1 |
|  | membrane part | 2 | 13 |
|  | organelle | 3 | 13 |
|  | organelle part | 1 | 5 |
| Molecular function | binding | 37 | 40 |
|  | catalytic activity | 34 | 43 |
|  | structural molecule activity | 1 | 9 |
|  | transporter activity | 1 | 11 |
|  | enzyme regulator activity | 1 | 0 |
|  | molecular transducer activity | 2 | 0 |
|  | nucleic acid binding transcription factor activity | 5 | 0 |
|  | protein binding transcription factor activity | 1 | 0 |

*Supplemental Table3*. The gene expression difference of *S. mutans-V. parvula* dual biofilm with and without 250μM copper ions

|  | GO term | No. of up gene  (*sm-vp 500 vs sm-vp 250*) | No. of down gene  (*sm-vp 500 vs sm-vp 250*) |
| --- | --- | --- | --- |
| Biological process | biological regulation | 14 | 4 |
|  | cellular component organization or biogenesis | 6 | 12 |
|  | cellular process | 41 | 50 |
|  | establishment of localization | 4 | 14 |
|  | localization | 4 | 14 |
|  | metabolic process | 50 | 55 |
|  | multi-organism process | 1 | 2 |
|  | regulation of biological process | 14 | 4 |
|  | response to stimulus | 3 | 5 |
|  | single-organism process | 28 | 40 |
|  | immune system process | 1 | 0 |
|  | signaling | 2 | 0 |
| Cellular component | cell | 23 | 32 |
|  | cell part | 23 | 32 |
|  | macromolecular complex | 7 | 17 |
|  | membrane | 4 | 17 |
|  | membrane-enclosed lumen | 1 | 1 |
|  | membrane part | 2 | 13 |
|  | organelle | 3 | 13 |
|  | organelle part | 1 | 5 |
| Molecular function | binding | 37 | 40 |
|  | catalytic activity | 34 | 43 |
|  | structural molecule activity | 1 | 9 |
|  | transporter activity | 1 | 11 |
|  | enzyme regulator activity | 1 | 0 |
|  | molecular transducer activity | 2 | 0 |
|  | nucleic acid binding transcription factor activity | 5 | 0 |
|  | protein binding transcription factor activity | 1 | 0 |

*Supplemental Table4.* The gene expression difference of *S. mutans-V. parvula* dual biofilm with 250μM copper ions and 500μM copper ions
